# Effectiveness of exercise intervention on children and adolescents with depression: a systematic review and meta-analysis of randomized controlled trial

**DOI:** 10.3389/fpsyt.2025.1699554

**Published:** 2025-11-04

**Authors:** Haoming Yan, Rui Chen, Daiwei Chen, Changdong Li

**Affiliations:** ^1^ School of Physical Education, Chengdu Sport University, Chengdu, China; ^2^ School of Physical Education and Sport Science, Fujian Normal University, Fuzhou, China; ^3^ Department of Physical Education, Zunyi Normal University, Zunyi, China

**Keywords:** exercise intervention, depression, children and adolescents, meta-analysis, systematic review

## Abstract

**Objective:**

The increasing occurrence of depression in children and teenagers has garnered significant social attention. Although many studies have explored the effect of exercise on alleviating depressive symptoms, substantial evidence regarding its efficacy in children and adolescents with clinical diagnoses of depression remains insufficient. This research performs a systematic review and meta-analysis aimed at assessing the true effectiveness of exercise interventions for this demographic.

**Methods:**

We searched five databases (PubMed, Embase, Cochrane, EBSCO, and Web of Science) for studies available up to August 18, 2025. All data analyses were conducted using Review Manager software. Four subgroup analyses were carried out according to exercise frequency, session length, duration of the intervention, and control group type in order to identify the sources of variability.

**Results:**

Following a thorough review of the 2,475 articles that were initially identified, a total of 15 studies were finally incorporated into the meta-analysis. These studies included 428 participants in the groups receiving exercise interventions and 403 participants in the control groups. The findings indicated that exercise produced a notable beneficial impact on children and adolescents suffering from depression (SMD = -1.14, 95% CI: -1.57 to -0.72, p < 0.001). Subgroup analyses further revealed that different weekly exercise frequencies, session durations, and intervention periods all demonstrated statistically significant beneficial effects on depressive symptoms. Moreover, exercise interventions achieved therapeutic effects comparable to those of conventional treatments.

**Conclusion:**

Exercise interventions were found to offer substantial therapeutic benefits for depressed children and adolescents in this study. Interventions performed more than three times per week, lasting less than 60 minutes per session, and sustained over eight weeks were found to be the most effective. Compared with traditional treatment approaches, exercise interventions achieved similarly positive outcomes. These findings provide strong evidence for optimizing exercise prescriptions and health management strategies for adolescent mental health. Educators, parents, and school administrators should incorporate age-appropriate physical activities into daily life and design exercise programs with suitable frequency, duration, and intensity.

**Systematic Review Registration:**

https://www.crd.york.ac.uk/PROSPERO/, identifier CRD420251121698.

## Introduction

The issue of depression in both children and adolescents has become a significant mental health challenge, carrying serious consequences for both individual growth and overall public health (1). The worldwide occurrence of depression in children and teenagers has been on the increase in recent years (2). Recent epidemiological data indicate that the prevalence of major depressive disorder in this population is approximately 3.7%, while the prevalence of depressive symptoms of any severity reaches 21.3%, and the prevalence shows an increasing trend with age (3, 4). Of particular concern is the occurrence of depression at an early age, as it is closely associated with declines in academic performance, challenges in peer and family relationships, increased social isolation, and a heightened risk of self-harm and suicide, which is one of the leading causes of death among adolescents worldwide (5, 6). Additionally, experiencing depression during these crucial developmental stages heightens the risk of future episodes and the emergence of other psychiatric disorders in later life, highlighting the immediate need for effective and feasible treatment options (7).

Treatment options currently available for addressing depression in children and adolescents primarily consist of psychotherapy and pharmacological approaches (8). Medications such as selective serotonin reuptake inhibitors (SSRIs) have demonstrated efficacy; however, they may raise concerns regarding potential side effects, including an increased risk of suicidal ideation in younger patients, which requires careful clinical monitoring (9). Non-pharmacological treatments are primarily psychotherapy, with approaches such as cognitive behavioral therapy and interpersonal therapy recognized as the main forms of treatment, though they are often resource- and time-intensive and unevenly distributed, particularly in low-resource or rural communities (10, 11). In addition, adherence to medication and engagement in therapy may be hindered by stigma, family attitudes, and motivational challenges that are common among adolescents with depression (12, 13). These barriers highlight the need for safe, affordable, and accessible complementary or alternative treatment options.

Exercise has come to be acknowledged more and more as a beneficial non-drug approach for addressing depression in young individuals and teenagers (14). Beyond its well-documented benefits for physical health, regular exercise may exert positive effects on mental health through multiple biological and psychosocial pathways. The enhancement of physiological processes involves the management of the hypothalamic-pituitary-adrenal (HPA) axis, the decrease of systemic inflammation, the encouragement of neuroplasticity, the production of brain-derived neurotrophic factor (BDNF), and the enhancement of sleep quality (15, 16). On a psychosocial level, exercise can strengthen self-esteem, provide a sense of achievement, reduce rumination, and promote social connectedness when conducted in group settings (17, 18). Evidence from adult populations demonstrates that structured exercise interventions can yield moderate to large reductions in depressive symptoms, suggesting that similar benefits may extend to younger populations (19).

Although previous studies have summarized the effects of exercise on depressive symptoms in children and adolescents, their populations often did not consist of individuals formally diagnosed with depression but instead included many participants with normal mental and psychological status. This undoubtedly weakens the interpretability of the results to a considerable extent and limits their generalizability to real-world applications (20, 21). Moreover, previous studies on children and adolescents have yielded inconsistent findings. For example, some meta-analyses have reported significant improvements in depressive symptoms following structured exercise programs (20, 22, 23), whereas other studies have found that not all forms of exercise produce significant effects, and some results have even been disappointing (24, 25). Such discrepancies not only reflect heterogeneity in study design, intervention modalities, and participant characteristics but also highlight that the existing evidence remains insufficient to provide strong support for clinical interventions in children and adolescents with diagnosed depression. While systematic reviews have highlighted the possibilities that exercise holds for treating depression, there remains a significant gap in thorough analyses aimed specifically at children and adolescents diagnosed with depression. Because the precise effects of exercise on this population are still unclear, a systematic review and meta-analysis are needed. Such an effort is necessary to evaluate the effectiveness of exercise interventions in a detailed and quantitative manner, ultimately furnishing strong evidence for evidence-based clinical practices and guiding future research initiatives.

The aim of this research is to conduct a comprehensive review and meta-analysis of randomized controlled trials evaluating the effects of exercise interventions on reducing depressive symptoms in children and adolescents diagnosed with depression. Through the synthesis of existing evidence, this research intends to establish the overall efficacy of exercise in this clinically validated group, thus offering evidence-based insights for treating depression in young people and guiding future research initiatives.

## Methods

### Registration

This systematic review has been officially registered with PROSPERO under the registration number CRD420251121698. In conducting this review, we have strictly followed the PRISMA guidelines, which stand for Preferred Reporting Items for Systematic Reviews and Meta-Analyses (26). Adhering to these established guidelines ensures that our review is comprehensive and maintains high standards in reporting, thus contributing to the overall credibility and reliability of our findings in the field of systematic reviews and meta-analyses.

### Search strategy

A thorough review of the literature was performed using Web of Science, EBSCO, PubMed, Embase, and the Cochrane Library, covering records from the beginning of these databases up until August 18, 2025. The search strategy was built on terms related to “exercise,” “depression,” “children,” “adolescents,” and “randomized controlled trial,” supplemented by synonyms and controlled vocabulary. To find any additional publications that met the eligibility standards for inclusion in our review, the reference lists of relevant research were carefully examined. This process of screening references allowed us to broaden our search and identify pertinent literature that may have initially been overlooked. The supplemental resources provide the specific search tactics that were used, which provide a thorough knowledge of our search approach.

### Eligibility criteria

The eligibility criteria were: (1) participants aged 5–19 years with a confirmed clinical diagnosis of depression (27); (2) the intervention groups included different types of exercise. As most studies aimed to achieve lasting improvements in participants, the focus was primarily on repeated exercise sessions conducted over several weeks, while the control groups included those receiving no exercise, maintaining their usual lifestyle, or receiving standard care (28); (3) randomized controlled trial (RCT) design; and (4) studies reporting outcomes related to changes in depressive symptom measures.

Studies were excluded if they: (1) were not randomized trials; (2) did not include a control group; (3) failed to report depression-related outcomes; or (4) were conference abstracts, theses, retrospective analyses, books, letters, or patents.

### Study selection and data extraction

The process of literature screening and data extraction was conducted independently by two researchers, with any disagreements addressed through consultation with a third researcher. To ensure that unrelated papers were excluded, titles and abstracts underwent evaluation during the initial screening phase. Subsequently, the full texts of the remaining articles were assessed, leading to the removal of those that failed to satisfy the established inclusion and exclusion criteria.

The collected information comprised the author’s name, publication year, number of participants, details of the interventions for both the experimental and control groups, depression diagnoses, as well as the intervention’s duration, frequency, intensity, and the tools utilized for evaluating depressive symptoms.

### Risk of bias assessment

In accordance with the recommendations provided in the Cochrane Handbook for Systematic Reviews of Interventions (version 2.0), two researchers independently evaluated the possibility of bias (29). Any differences in their evaluations were addressed by conferring with a third researcher.

### Statistical analysis

Review Manager was used to do statistical analyses of continuous variables. Depending on the measurement methods and units used, effect sizes were shown as either weighted mean differences (WMD) or standardized mean differences (SMD) (30). Heterogeneity across studies was evaluated with the I² statistic. A fixed-effect model was applied when heterogeneity was low (p < 0.1 and I² < 50%), whereas a random-effects model was used in other cases. Sensitivity analyses were conducted employing the leave-one-out methodology (31); if the combined outcomes varied significantly following the exclusion of a study, it was deemed that the particular study had a considerable impact.

## Results

### Literature search results

**Figure 1:**
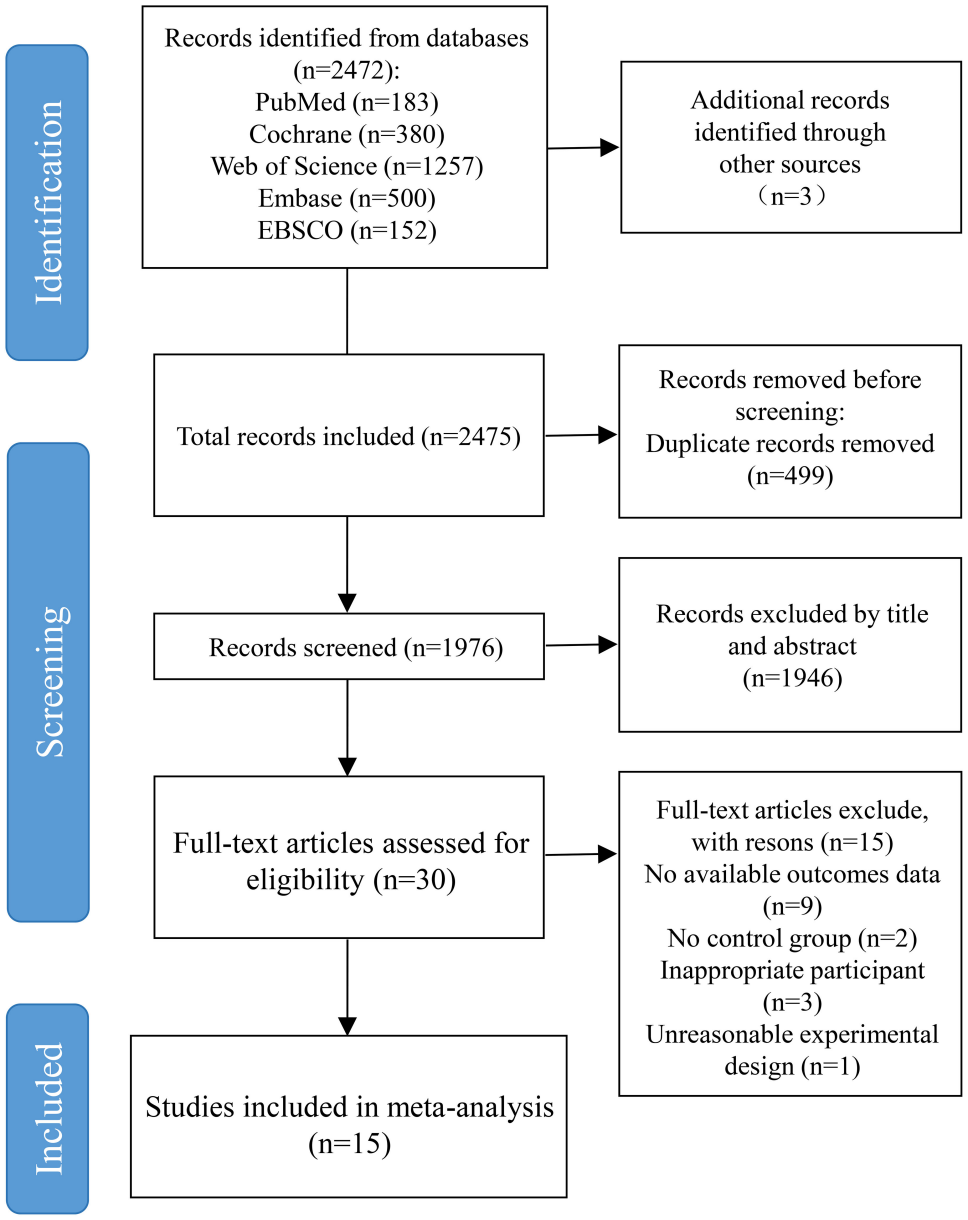
Flow diagram of literature selection process.

Initially, 2,475 records were identified in the databases, and 499 duplicates were eliminated. The process of examining titles and abstracts led to the removal of 1,946 entries. Subsequently, a comprehensive assessment of full texts resulted in the dismissal of 15 additional studies that failed to satisfy the eligibility requirements. Ultimately, 15 studies were deemed eligible and incorporated into the final analysis. **Figure 1** illustrates the selection process.

### Basic characteristics of the included literature

This review incorporated fifteen randomized controlled trials (32–46) published between 2005 and 2024. Altogether, 428 participants underwent exercise interventions, while 403 served as controls, all diagnosed with depression of varying severity. Aerobic exercise was the predominant intervention. Intervention periods ranged from 5 to 16 weeks, with most extending beyond 6 weeks. In over half of the studies, exercise was performed at least three times weekly, and each session exceeded 30 minutes. Depression outcomes were assessed using established scales commonly applied in children and adolescents, including the SCL-90-R, CES-D, and BDI. The key features of the included studies are shown in **Table 1**.

**Table 1:** Characteristics of eligible studies.

Included studies	Sample size (N)	Age of participants	Intervention		Diagnosis	Dose	Outcome measures
			E	C			
Jeong 2005, RCT (32)	E=20, C = 20	EG: 16 CG: 16	Group dancing	Normal lifestyle	Mild depression	12 weeks, 45-min/session, 3 times/week	SCL-90-R
Nabkasorn 2006, RCT (33)	E=21, C = 28	18.8 ± 0.7	Group jogging	Non-exercise	Mild to moderate depressive symptoms	8 weeks, 50-min/session, 5 times/week	CES-D
Mohammadi 2011, RCT (34)	E=40, C = 40	15-18	Ball sports	Non-exercise	Ranging from mild to severe depression	8 weeks, 75-min/session, 3 times/week	BDI
Hughes 2013, RCT (35)	E=14, C = 12	EG: 17 CG: 17	Aerobic exercise and strength training	Usual treatment	Depression	12 weeks, 30-min/session, 3 times/week	CDRS-R
Carter 2015, RCT (36)	E=25, C = 18	EG: 15.4 ± 1.0 CG: 15.4 ± 0.9	Aerobic exercise and strength training	Usual treatment	Depression	6 weeks, 60-min/session, 2 times/week	CDI-2
Turner 2017, RCT (37)	E=44, C = 42	EG: 15.4 CG: 15.4	Circuit training	Usual treatment	Depression	6 weeks, 60-min/session, 2 times/week	CDI-2
Wunram 2018, RCT (38)	E=35, C = 17	EG: 16.0 ± 1.2 CG: 15.7 ± 1.1	Bicycle practice	Usual treatment	Depression	6 weeks, 30-min/session, 4 times/week	DIKJ
Zhang 2018, RCT (39)	E=32, C = 30	18.4 ± 2.0	Tai Chi	Normal lifestyle	Mild depression	8 weeks, 90-min/session, 2 times/week	PHQ-9
Silva 2020, RCT (40)	E=10, C = 10	EG: 12 ± 1 CG: 12 ± 1	Swimming	Non-exercise	Depression	8 weeks, 45-min/session, 2 times/week	CDI
Lin 2020, RCT (41)	E=21, C = 18	EG: 12.7 ± 0.7 CG: 12.6 ± 0.5	Running and walking	Usual treatment	Mild depression	12 weeks, 30-min/session, 4 times/week	PHQ-9
Zhang 2021, RCT (42)	E=66, C = 69	14.3 ± 1.8	Running	Drug therapy	Depression	16 weeks, 30-min/session, 3 times/week	HAMD-24
Philippot 2022, RCT (43)	E=20, C = 20	EG: 15.5 ± 1.8 CG: 15.2 ± 1.5	Group games and strength training	Social relationship therapy	Depression	5 weeks, 60-min/session, 4 times/week	HADS-D, HAM-D and CDI
Seddigh 2023, RCT (44)	E=31, C = 31	EG: 14.1 ± 1.1 CG: 14 ± 1.0	Yoga	Routine care	Depression	8 weeks, 60-min/session, 1 times/week	CDI
Uebelacker 2023, RCT (45)	E=21, C = 21	15.0 ± 1.5	Yoga	CBT	Depression	12 weeks, 45-min/week	QIDS-A-CR and BDI
Gu 2024, RCT (46)	E=28, C = 27	16.7 ± 0.4	Ball sports	Normal lifestyle	Mild depression	8 weeks, 60-min/session, 3 times/week	CES-D and BDI

E, experimental group; C, control group; SCL-90-R, the Symptom Check List-90-Revision; CES-D, the Centre for Epidemiologic Studies Depression; BDI, Beck Depression Inventory; CDRS-R:Childhood Depression Rating Scale-Revised; CDI-2, the Children’s Depression Inventory; DIKJ, Depressions Inventar für Kinder und Jugendliche; PHQ-9, Patient Health Questionnaire depression scale; CDI, Child Depression Inventory; HAMD-24, Hamilton Depression Scale 24; HADS-D, the Hospital Anxiety & Depression Scale; HAM-D, Hamilton Rating Scale for Depression; QIDS-A-CR, Quick Inventory of Depression–Adolescent Version—Clinician Rating; CBT, cognitive-behavioral therapy.

### Risk of bias

**Figure 2:**
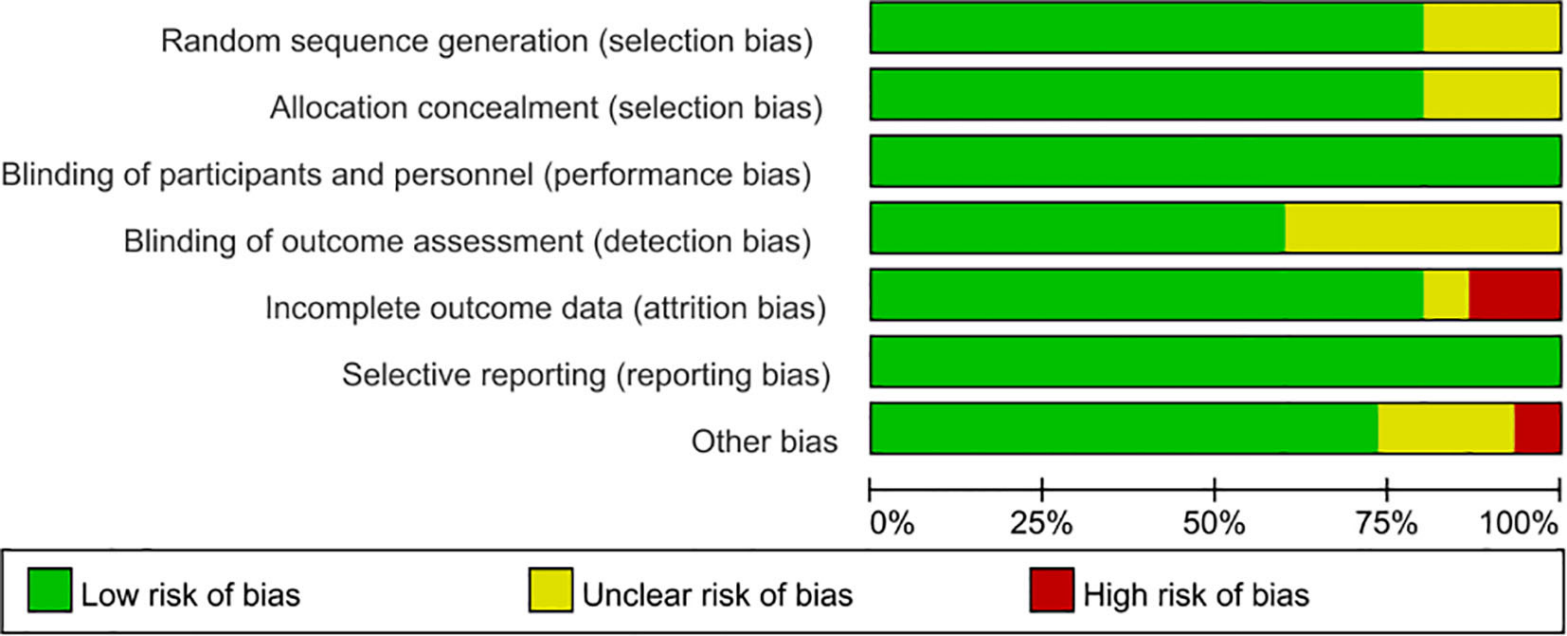
Overall overview graph of bias risk in included studies.

**Figure 3:**
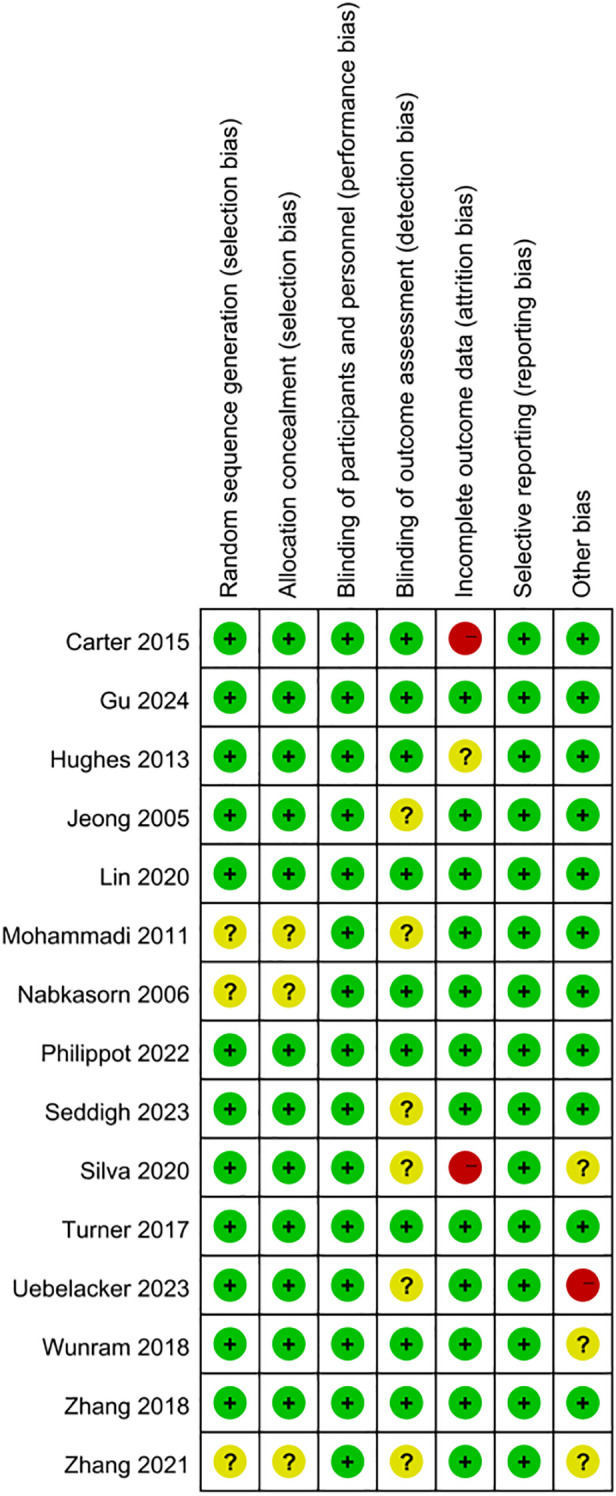
Risk of bias evaluation graph for the included literature.

**Figures 2** and **3** present assessments of bias risk, both overall and for individual studies. Just three studies were categorized as high risk, particularly concerning the areas of “incomplete outcome data” and “other potential biases.” Given the intrinsic nature of exercise interventions, it was deemed unfeasible to blind participants and personnel; therefore, every study received a low risk rating in this regard. Overall, the included trials demonstrated satisfactory methodological quality and provided reliable evidence for this review.

### Meta-analysis

#### Overall effectiveness

**Figure 4:**
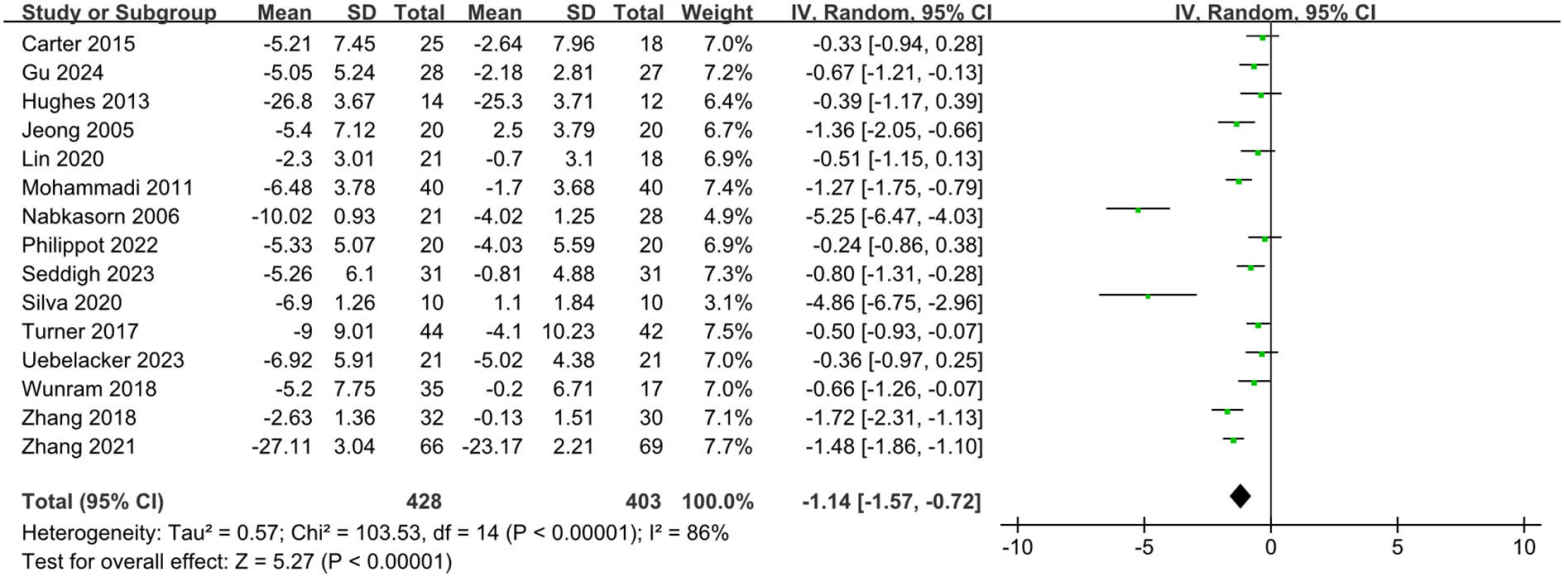
Forest plot of the overall efficacy of exercise intervention on depression.

Exercise substantially decreased depression symptoms in children and adolescents when compared to controls, according to a meta-analysis using a random-effects model (SMD = -1.14, 95% CI: -1.57 to -0.72, p < 0.001) (**Figure 4**). Subgroup analyses were conducted to determine possible sources and contributing variables because of the significant heterogeneity among the included studies (I^2^ = 86%).

### Subgroup analysis

#### Intervention frequency

**Figure 5:**
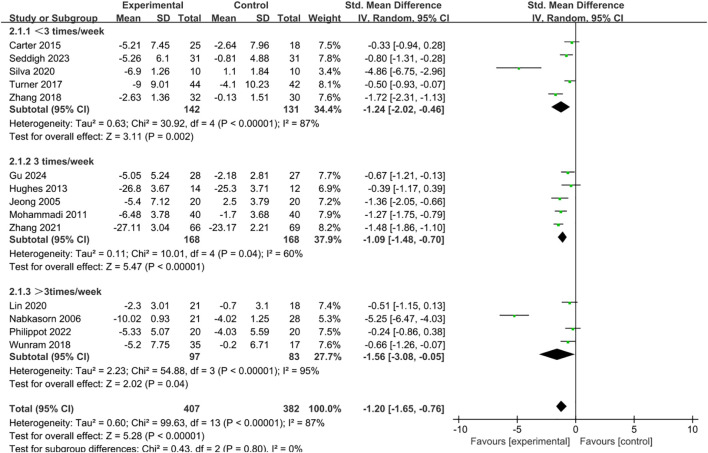
Forest plot of subgroup analysis by intervention frequency.

Intervention frequency was divided into three categories: less than three sessions per week, three sessions per week, and more than three sessions per week. The subgroup analysis demonstrated significant improvements in depressive symptoms across all three categories (**Figure 5**). Among them, interventions performed more than three times weekly (SMD = -1.56, 95% CI = -3.08 to -0.05, p = 0.04) produced slightly stronger effects than those delivered less than three times weekly (SMD = -1.24, 95% CI = -2.02 to -0.46, p = 0.002) or exactly three times weekly (SMD = -1.09, 95% CI = -1.48 to -0.70, p < 0.001).

#### Duration of single intervention

**Figure 6:**
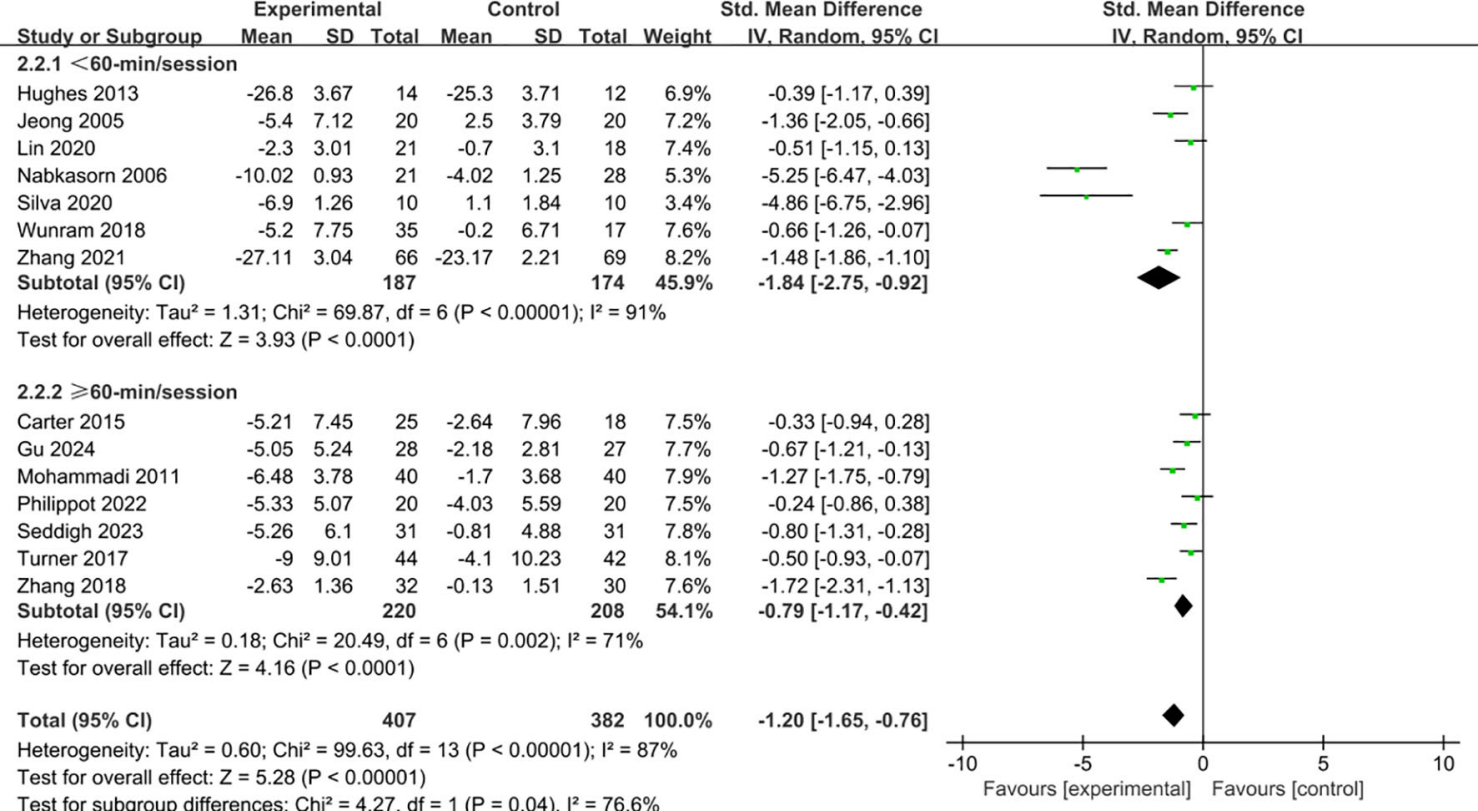
Forest plot of subgroup analysis by duration of single intervention.

The length of each intervention session was categorized into two groups: less than 60 minutes and 60 minutes or more (including 60 minutes). Analysis of subgroups indicated that both classifications were linked to noteworthy decreases in depressive symptoms. (**Figure 6**). Nevertheless, interventions lasting under 60 minutes (SMD = -1.84, 95% CI = -2.75 to -0.92, p < 0.001) produced a stronger therapeutic effect than those lasting 60 minutes or longer (SMD = -0.79, 95% CI = -1.17 to -0.42, p < 0.001).

#### Intervention cycle

**Figure 7:**
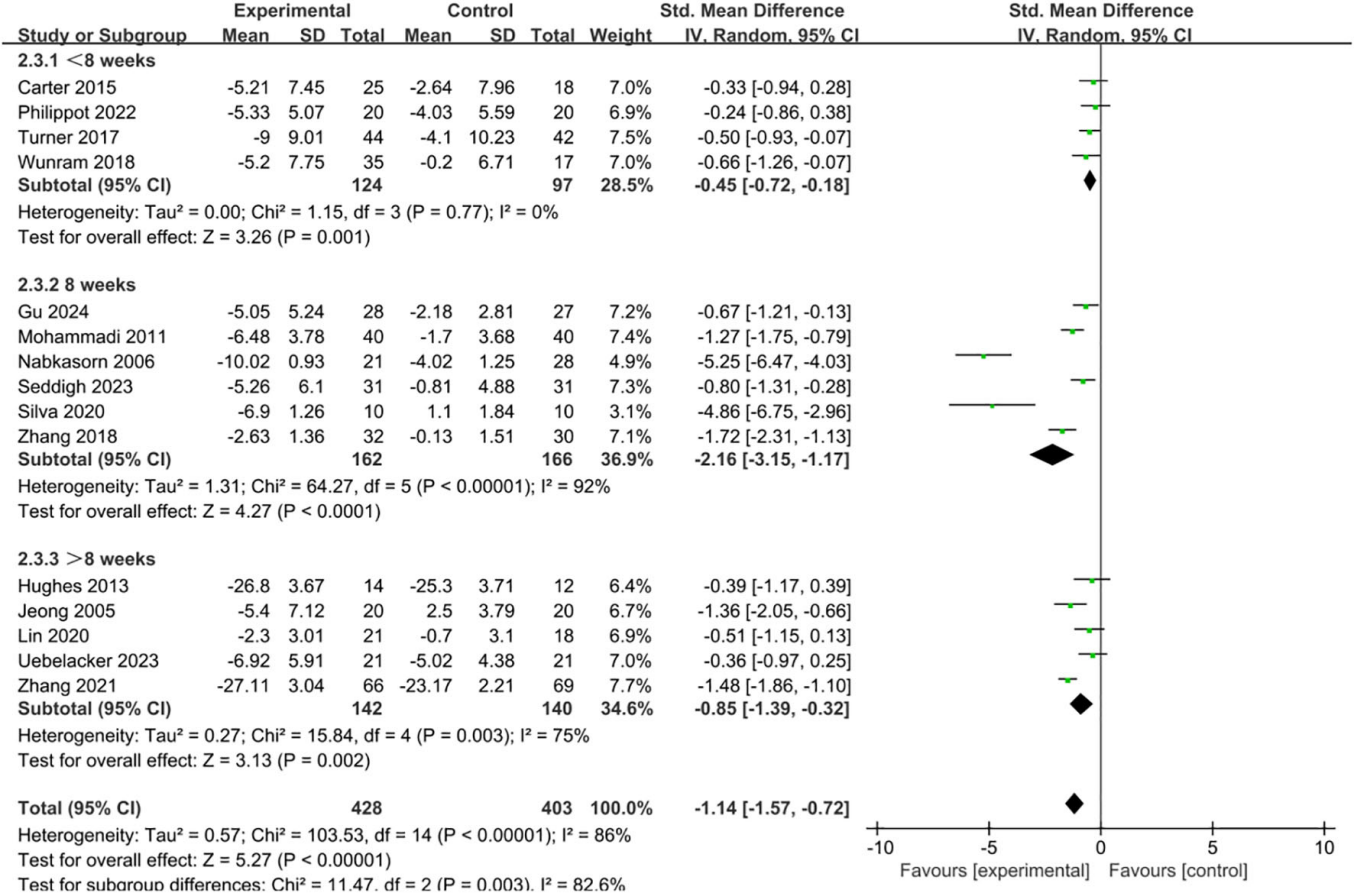
Forest plot of subgroup analysis by intervention cycle.

The intervention duration was categorized into three groups: less than 8 weeks, exactly 8 weeks, and more than 8 weeks. Subgroup analysis revealed that all three durations were significantly effective in alleviating depressive symptoms (**Figure 7**). Notably, an 8-week program (SMD = -2.16, 95% CI = -3.15 to -1.17, p < 0.001) demonstrated the greatest efficacy, surpassing both programs longer than 8 weeks (SMD = -0.85, 95% CI = -1.39 to -0.32, p = 0.002) and those shorter than 8 weeks (SMD = -0.45, 95% CI = -0.72 to -0.18, p = 0.001).

#### Control group category

**Figure 8:**
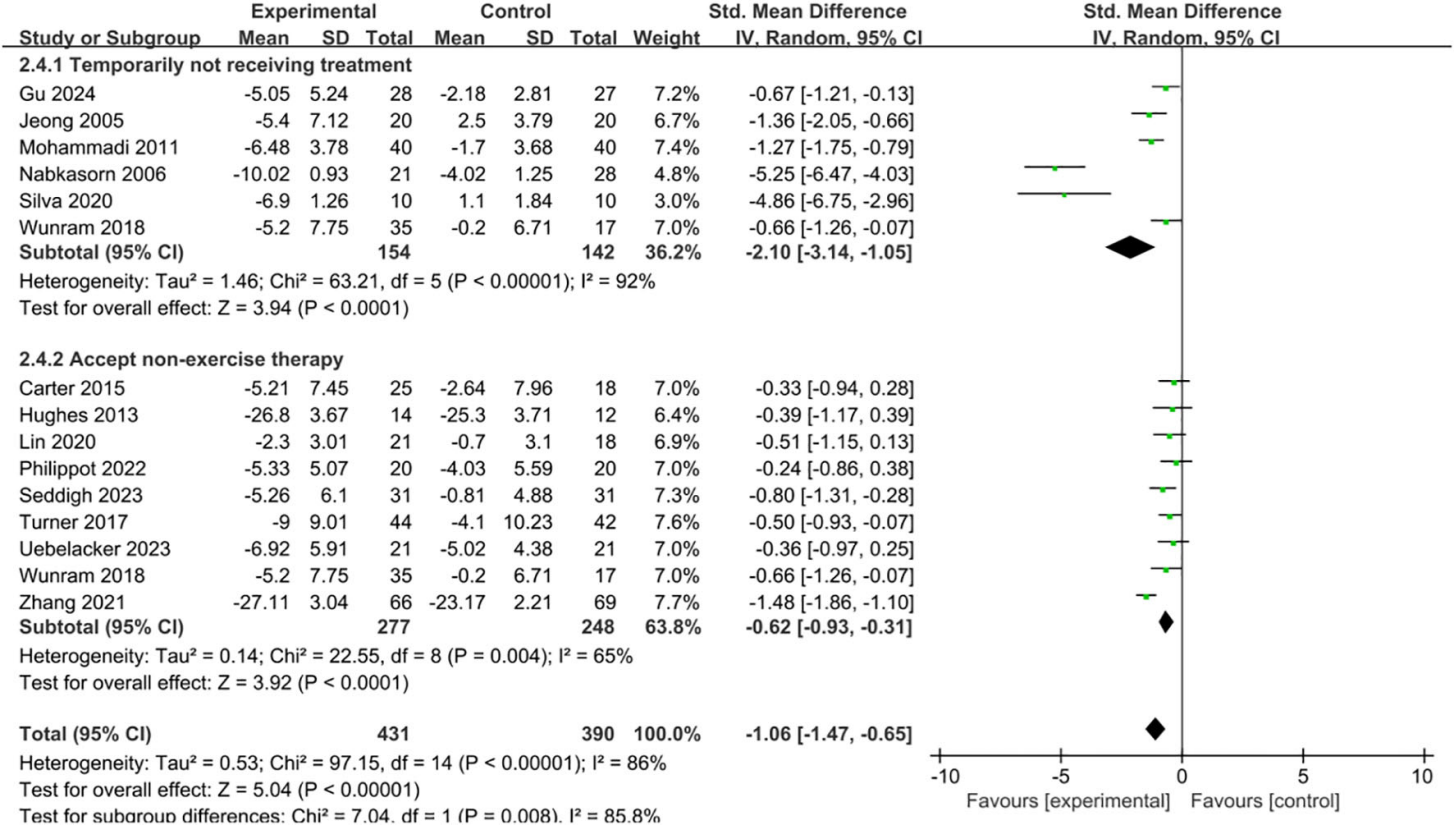
Forest plot of subgroup analysis by control group type.

The control groups were categorized into two types: those temporarily not receiving treatment and those receiving non-exercise therapies. Subgroup analysis showed that, compared with the control groups, both categories of intervention groups produced significant improvements in depressive symptoms (**Figure 8**). Specifically, interventions in the no-treatment group (SMD = -2.10, 95% CI = -3.14 to -1.05, p < 0.001) demonstrated greater efficacy than those in the non-exercise therapy group (SMD = -0.62, 95% CI = -0.93 to -0.31, p < 0.001).

#### Exercise intervention model

**Figure 9:**
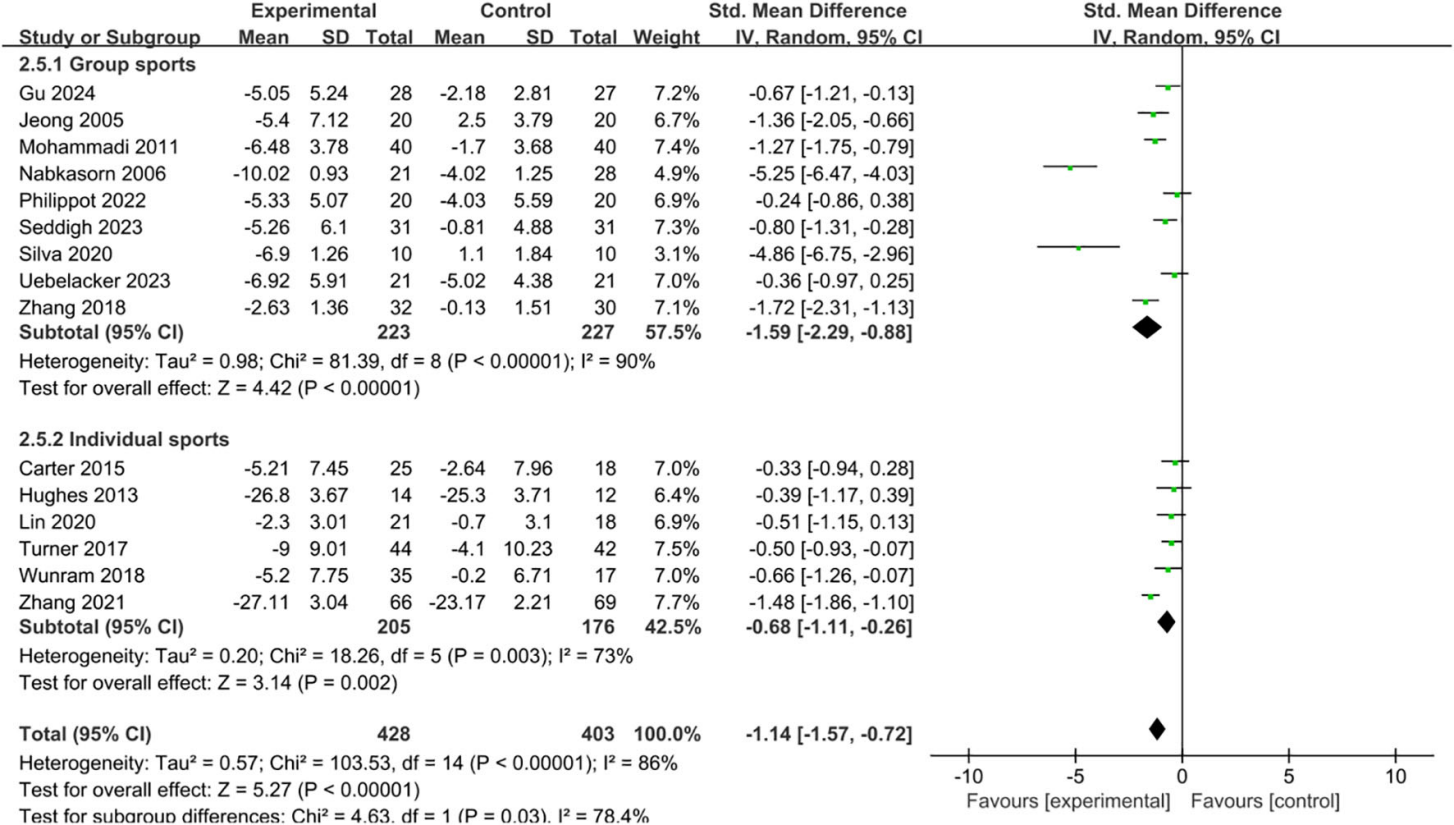
Forest plot of subgroup analysis by exercise intervention model.

The study categorized exercise therapy into two types: group-based and individual-based. Both types effectively alleviated depressive symptoms (**Figure 9**). However, group sports had a markedly and substantially greater therapeutic effect than (SMD = -1.59, 95% CI = -2.29 to -0.88, p < 0.001) than individual sports (standardized mean difference = -0.68, 95% CI = -1.11 to -0.26, p = 0.002).

### Sensitivity analysis and publication bias

**Figure 10:**
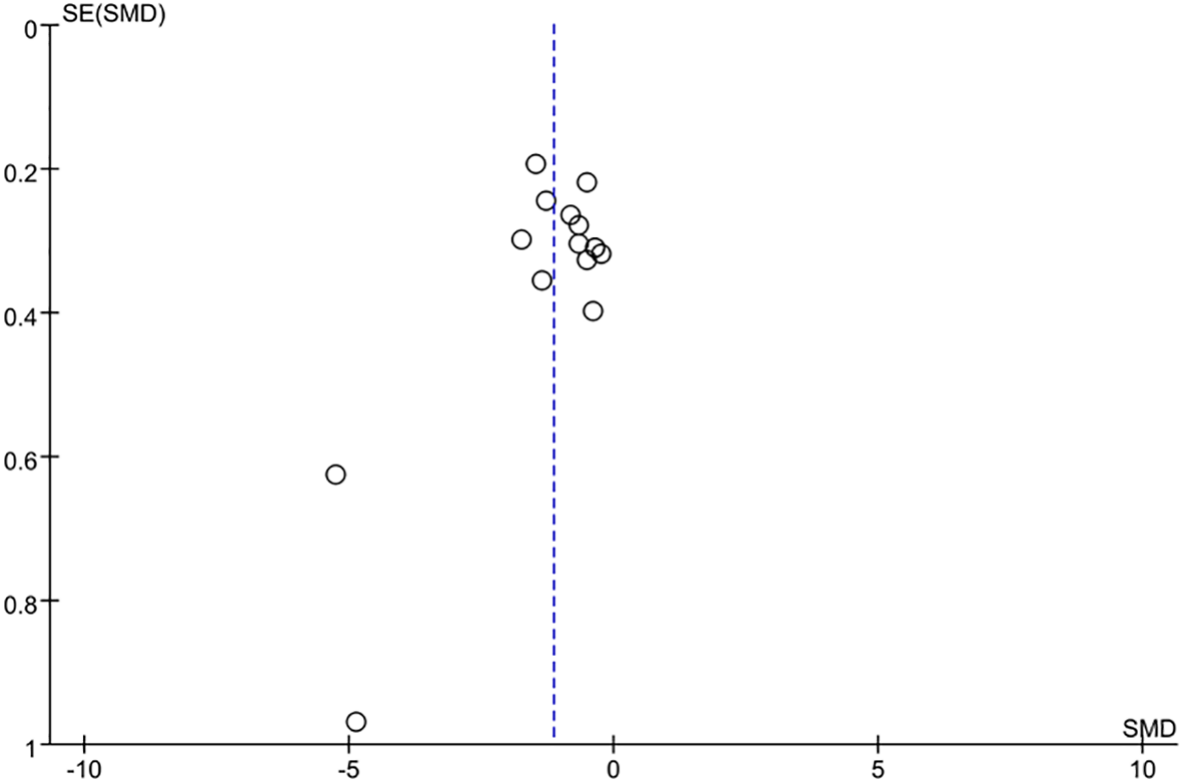
Publication bias analysis results.

The sensitivity analysis performed with the leave-one-out approach revealed that the overall effect size remained stable and did not show statistically significant variations when any individual study was omitted, highlighting a strong consistency in the findings. A funnel plot was used to evaluate potential publication bias, and the results showed a mostly symmetrical distribution with a few dispersed spots (**Figure 10**). Furthermore, p > 0.05 from the Egger’s test indicated that there was no proof of publication bias (47).

## Discussion

This meta-analysis and systematic review, which focused on randomized controlled trials (RCTs), assessed the impact of exercise interventions on children and adolescents who have been diagnosed with depression. Rigorous inclusion criteria guaranteed that all participants possessed a confirmed diagnosis of depression. The findings revealed the therapeutic benefits of exercise for this population, addressed existing research gaps, and further validated the generalizability of exercise as a treatment for depression.

The findings provide compelling evidence for the beneficial therapeutic effects of exercise by showing that it can considerably reduce depression symptoms in this population. This conclusion aligns with earlier research results and further strengthens the significance of exercise as a powerful non-drug intervention. Notably, substantial evidence from neuroscience and physiology offers a solid foundation for explaining the antidepressant effects of exercise interventions (21, 22). Initially, engaging in physical activity stimulates the release and rebalancing of various neurotransmitters within the central nervous system, such as serotonin (5-HT), dopamine (DA), and norepinephrine (NE) (48). These neurochemicals are essential for mood regulation, the mediation of reward processes, and the body’s response to stress (49). Low levels of 5-HT and DA are often associated with depressive symptoms, whereas exercise increases their concentrations, helping to enhance emotional stability and motivation. Second, it has been shown that exercise promotes the release of BDNF, or brain-derived neurotrophic factor (50). This essential chemical promotes neuroplasticity and the development of new neurons, which greatly aids in learning, memory, and emotional regulation (51). A recent analysis shows that engaging in physical activity leads to lower levels of catabolic cortisol and enhances neuroplasticity within specific brain regions. This phenomenon correlates with advancements in synaptogenesis, neurogenesis, the release of neurotrophic factors, as well as various neuroendocrine modifications. Such changes might be associated with improvements in cognitive and emotional processes, potentially resulting in a decrease in psychosocial stress symptoms over time (52). Moreover, disruptions in circadian rhythms are recognized as a significant contributor to depression. Research indicates that regular physical exercise positively impacts the organization and growth of the brain, aiding in its ability to adjust the circadian rhythm in response to external temporal signals (53). Additionally, exercise can influence the function of the hypothalamic-pituitary-adrenal (HPA) axis, which is closely linked to the symptoms of major depression (54, 55). Hyperactivation of the HPA axis leads to chronically elevated cortisol levels, which is one of the major mechanisms underlying the onset and persistence of depression (56). Changes in cortisol levels are conducive to enhancing cognitive abilities, helping children and adolescents improve their academic performance and develop a sense of academic self-efficacy (57). Moreover, they also contribute to reducing physiological stress load and promoting homeostatic balance (58). Research has indicated that both lower and higher levels of exercise intensity can enhance neurogenesis. Nevertheless, exercise of higher intensity tends to produce more significant positive impacts on neurocognitive function, primarily due to its association with increased testosterone release, which is strongly linked to cognitive performance (59, 60). It is worth noting that some studies have reported no conclusive interaction between aerobic exercise and BDNF or cortisol. Nevertheless, these findings acknowledge that such inconsistencies may result from methodological differences in exercise type, duration, prescribed intensity, and frequency, which further underscores the importance of scientifically designing exercise programs (61). Meanwhile, exercise exerts beneficial effects on other bodily functions closely related to depression. For example, regular exercise improves sleep architecture and sleep quality, while sleep disturbances are both an important trigger and a sustaining factor of depression (62, 63). Exercise also enhances immune system functioning and reduces chronic inflammatory responses (64). There is mounting evidence that the severity of depressive symptoms is strongly associated with elevated levels of inflammatory markers, such as TNF-α and IL-6. In contrast, engaging in exercise can reduce these inflammatory markers, leading to antidepressant effects (65). However, it should be noted that not all forms of exercise lead to significant improvements in individual depression control. A systematic review indicated that even after engaging in various types of chronic exercise interventions, the participating children and adolescents gained only limited benefits in terms of depression regulation (66). Therefore, when using exercise as a strategy to alleviate depressive symptoms, it may be advisable to avoid employing different forms of chronic exercise.

The subgroup analysis of intervention frequency showed that exercise performed less than three times, exactly three times, or more than three times per week was effective in reducing depressive symptoms, with the best outcomes observed at a frequency of more than three sessions per week, followed by less than three sessions per week. This finding indicates that exercise exhibits a “more is better” dose–response relationship within a certain range, which is consistent with previous research findings (49, 67). Higher-frequency interventions may more fully exert exercise’s neuroregulatory and psychological benefits, producing positive physiological and psychological effects and leading to greater symptom improvement in the short term (68). At the same time, low-frequency exercise interventions also showed positive effects, likely due to their greater feasibility and adherence (69). For children and adolescents, lower-frequency programs are easier to integrate into academic and daily routines, reducing additional time burdens and supporting long-term adherence, thereby providing a stable means of emotional regulation (70). Therefore, exercise prescriptions should strike a balance between “maximizing effectiveness” and “ensuring sustainability,” and be tailored to individual needs. Such programs can be more easily integrated into the daily lives of this population as part of the curriculum or as extracurricular physical activity initiatives (71). For those with strong motivation or more severe depressive symptoms, more than three sessions per week can be recommended, whereas for adolescents with lower adherence or heavy academic workloads, even about two regular sessions per week can still yield significant benefits (72).

The subgroup analysis of intervention duration showed that both sessions shorter than 60 minutes and those lasting 60 minutes or longer significantly alleviated depressive symptoms, but shorter sessions proved more effective. This result is consistent with previous findings (73). Exercise duration is not necessarily “the longer, the better”; rather, moderate sessions within a certain range may be more advantageous (74). First, shorter exercise sessions can effectively stimulate the release of neurotransmitters and emotion-regulating molecules while avoiding the fatigue and physical burden associated with prolonged activity (75, 76). For example, sudden excessive exercise may trigger stress responses, lactic acid accumulation, and elevated cortisol levels, which could in turn diminish the antidepressant effects of exercise (77). In contrast, sessions kept within 60 minutes are more likely to maintain positive emotional experiences and physiological adaptability, thereby promoting mental health more consistently (78). Second, shorter sessions are more easily accepted and adhered to by children and adolescents. For this population, prolonged exercise may increase feelings of boredom and fatigue or conflict with busy academic and daily schedules, leading to reduced compliance. By comparison, moderate durations of 30 to 60 minutes provide a sufficient exercise dosage while remaining manageable, making them better suited to the psychological characteristics and lifestyle patterns of students in school (79). Studies have shown that the immediate positive emotional experience during exercise is an important driving force for adolescents’ adherence, and that a moderate duration of exercise is more conducive to sustaining this positive experience (80).

The subgroup analysis of intervention duration indicated that short-term (<8 weeks), medium-term (=8 weeks), and long-term (>8 weeks) exercise interventions all effectively alleviated depressive symptoms, with the most significant effects observed in the medium-term interventions. This may be explained by several factors. On the one hand, medium-term interventions may coincide with the psychological and behavioral adaptation window (81). In the initial weeks of the intervention, individuals are primarily in an adjustment phase, and the emotional improvements brought about by exercise may not yet be stable. When the intervention extends beyond eight weeks, although positive effects may still occur, factors such as diminished novelty, reduced adherence, or conflicts with academic and daily demands may weaken its overall effectiveness (82, 83). Therefore, a duration of around eight weeks may achieve a relative balance between these factors, thereby producing the most favorable outcomes. On the other hand, the development of healthy behaviors requires a certain amount of time. A cycle of about two months is sufficient for adolescents to gradually establish regular exercise routines and experience tangible physical and psychological benefits, thereby enhancing self-efficacy and motivation to persist (84, 85). By comparison, too short an intervention period may be insufficient to build such habits, while an overly long duration may lead to diminished motivation or external disruptions, making adherence more difficult and consequently reducing effectiveness (18).

Examination of the control groups within each subgroup showed that the no-exercise group exhibited a greater improvement in depressive symptoms compared with the routine treatment group. This result not only supports the efficacy of exercise as a standalone intervention but also highlights its considerable potential for tackling depression in children and adolescents, consistent with previous research findings (86, 87). Although conventional treatments such as medication and psychotherapy remain the primary approaches for depression, they are often accompanied by certain side effects, limited accessibility, or heavy economic burdens (88). Exercise interventions, while improving depressive symptoms, bring almost no additional medication-related side effects. Based on the study data, it can be concluded that an appropriately designed exercise intervention demonstrates significant practical value in improving depressive symptoms, offering dual advantages of cost-effectiveness and universality. It can serve as an effective complement to traditional therapies. This finding is particularly relevant in regions with limited medical resources, providing a new adjunctive treatment approach for the underage population. From an integrated intervention perspective, exercise can complement psychotherapy or pharmacological treatments. Studies have demonstrated that exercise not only boosts mood directly but also raises self-esteem, enhances sleep quality, and strengthens cognitive performance, thus increasing the efficacy of traditional therapies (89). This suggests that exercise interventions may serve as an important catalyst within the traditional treatment system.

The analysis of subgroup variations regarding exercise intervention types indicated that both group and individual sports were effective in alleviating depressive symptoms; however, group sports yielded a notably stronger positive impact. This discovery indicates that social interaction might significantly contribute to the antidepressant effects of exercise interventions. Participating in group exercise not only offers the physical advantages associated with activity but also promotes emotional connections and friendships among participants, thereby enhancing social support and creating a sense of community that improves therapeutic results (90). For young people, taking part in group exercise in a secure and encouraging social setting can boost self-esteem, lessen feelings of isolation, and enhance emotional regulation, resulting in more successful reduction of depressive symptoms (91). Moreover, engaging in group exercise typically entails collaboration, rivalry, and the attainment of goals—experiences that have the potential to enhance self-efficacy and foster a sense of achievement, thereby encouraging positive emotional states (92). While solitary exercise also plays a role in mood regulation, the absence of social interaction might restrict emotional backing and outside motivation, leading to a somewhat reduced effectiveness. Consequently, when creating exercise intervention strategies, it is recommended to focus on activities that are group-oriented, such as aerobics sessions, team sports, or training in small groups, in order to fully leverage the additional psychological advantages that come from social support and group dynamics.

Our study revealed the intervention effects of exercise on children and adolescents with depression. It provided an in-depth discussion of the efficacy and possible mechanisms across different intervention frequencies, session durations, intervention periods, and control group types. This study, however, does have particular limitations. First, the intervention characteristics among the included studies were not fully standardized. Differences existed in exercise intensity, duration, and frequency, which may have contributed to the observed heterogeneity and affected the pooled results. Although we conducted subgroup analyses to minimize this influence, the diversity in intervention designs still poses challenges for interpreting the overall effect. Furthermore, restricting the included studies to English-language peer-reviewed publications may have introduced language and publication bias, since studies in other languages or local journals that reported null or less favorable results might have been overlooked. This limitation may slightly reduce the comprehensiveness of our findings. In addition, although we prioritized studies with higher methodological quality during the screening process, the methodological risks present in some of the included studies still, to some extent, affected the credibility of the clinical evidence, thereby reducing the level of confidence in the study conclusions. Finally, some studies involved relatively small sample sizes, which may have methodological limitations and restrict the generalizability of the findings. In future studies, we plan to investigate and compare different types of exercise interventions, conducting multicenter randomized controlled trials and long-term follow-ups to clarify the differences among various exercise modalities in alleviating depressive symptoms, maintaining long-term efficacy, and improving psychosocial functioning. We also intend to include studies published in multiple languages to expand the search scope, reduce potential bias, and provide more comprehensive insights.

## Conclusion

The results of this study indicate that exercise interventions have significant therapeutic effects for children and adolescents with depression. Appropriate exercise frequencies are effective, with the best outcomes observed at more than three sessions per week. Sessions lasting less than 60 minutes were more effective than those lasting 60 minutes or longer. The eight-week intervention produced the most favorable results. Compared with traditional treatment methods, exercise interventions have achieved similarly positive effects. These findings provide strong evidence for optimizing exercise prescriptions and health management strategies for adolescent mental health. Educators, parents, and school administrators should incorporate age-appropriate physical activities into daily life and develop exercise programs with suitable frequency, duration, and intervention periods.

## Data Availability

The original contributions presented in the study are included in the article/**Supplementary Material**. Further inquiries can be directed to the corresponding author.
