# Supplementary material for: Effectiveness of exercise intervention on children and adolescents with depression: a systematic review and meta-analysis of randomized controlled trial
Source: Front Psychiatry. 2025 Nov 4;16:1699554. doi: 10.3389/fpsyt.2025.1699554 (PMC12624221; doi:10.3389/fpsyt.2025.1699554)
Supplement: Supplementary file 1 [file DataSheet2.zip › Retrieve records/文字文稿2.docm]

**Pubmed**

(((((((("Exercise"[Mesh]) OR ("Resistance Training"[Mesh])) OR ("Tai Ji"[Mesh])) OR ("Qigong"[Mesh])) OR ("Yoga"[Mesh])) OR (((((((((exercises[Title/Abstract]) OR (physical activity[Title/Abstract])) OR (Aerobic Exercise[Title/Abstract])) OR (strength training[Title/Abstract])) OR (walk*[Title/Abstract])) OR (swim*[Title/Abstract])) OR (baduanjin[Title/Abstract])) OR (pilate[Title/Abstract])) OR (running[Title/Abstract]))) AND ((("Child"[Mesh]) OR ("Adolescent"[Mesh])) OR ((((children[Title/Abstract]) OR (teen*[Title/Abstract])) OR (adolesecnt*[Title/Abstract])) OR (youth*[Title/Abstract])))) AND (("Depression"[Mesh]) OR (((((Depressive Symptoms[Title/Abstract]) OR (Depressive Symptom[Title/Abstract])) OR (Emotional Depression[Title/Abstract])) OR (Symptom, Depressive[Title/Abstract])) OR (Depression, Emotional[Title/Abstract])))) AND (((randomized controlled trial[Title/Abstract]) OR (randomized[Title/Abstract])) OR (placebo[Title/Abstract]))

Cochrone

EMBASE

('exercise'/exp OR 'resistance training'/exp OR 'tai chi'/exp OR 'yoga'/exp) AND ('child'/exp OR 'adolescent'/exp OR 'children':ab,ti OR 'teen*':ab,ti OR 'adolesecnt*':ab,ti OR 'youth*':ab,ti) AND ('depression'/exp OR 'depressive symptoms':ab,ti OR 'depressive symptom':ab,ti OR 'emotional depression':ab,ti OR 'symptom, depressive':ab,ti OR 'depression, emotional':ab,ti) AND ('randomized controlled trial':ab,ti OR 'randomized':ab,ti OR 'placebo':ab,ti)
