# Supplementary figures and images for: Effectiveness of exercise intervention on children and adolescents with depression: a systematic review and meta-analysis of randomized controlled trial
Source: Front Psychiatry. 2025 Nov 4;16:1699554. doi: 10.3389/fpsyt.2025.1699554 (PMC12624221; doi:10.3389/fpsyt.2025.1699554)

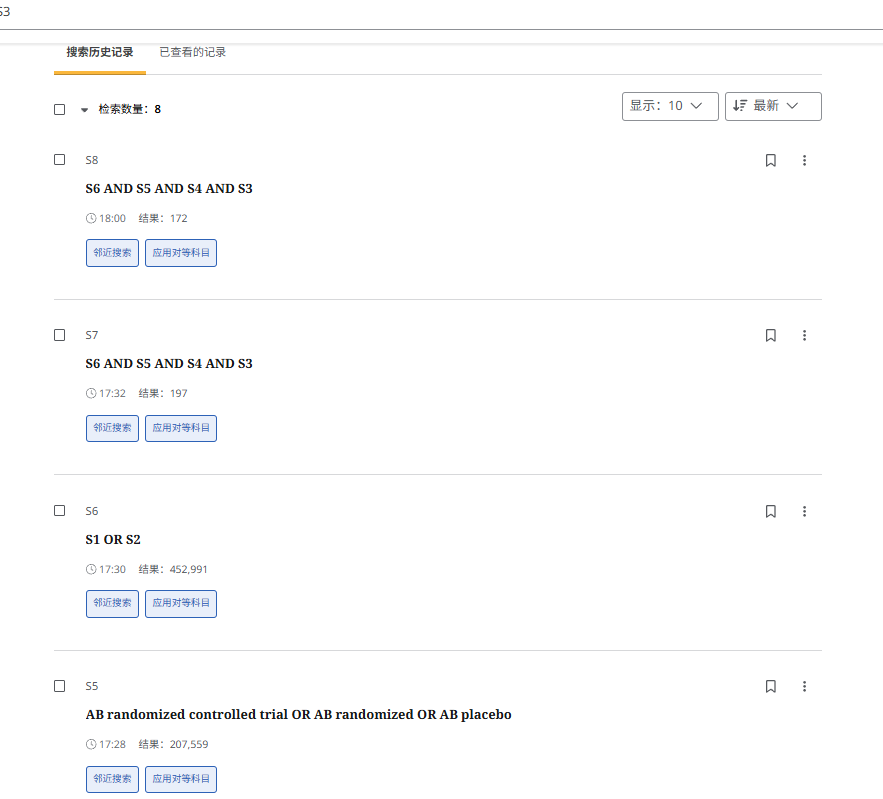

Supplement: Supplementary file 1 [file DataSheet2.zip › Retrieve records/17d673e0-f582-44bb-99a6-991bde257f78.png]

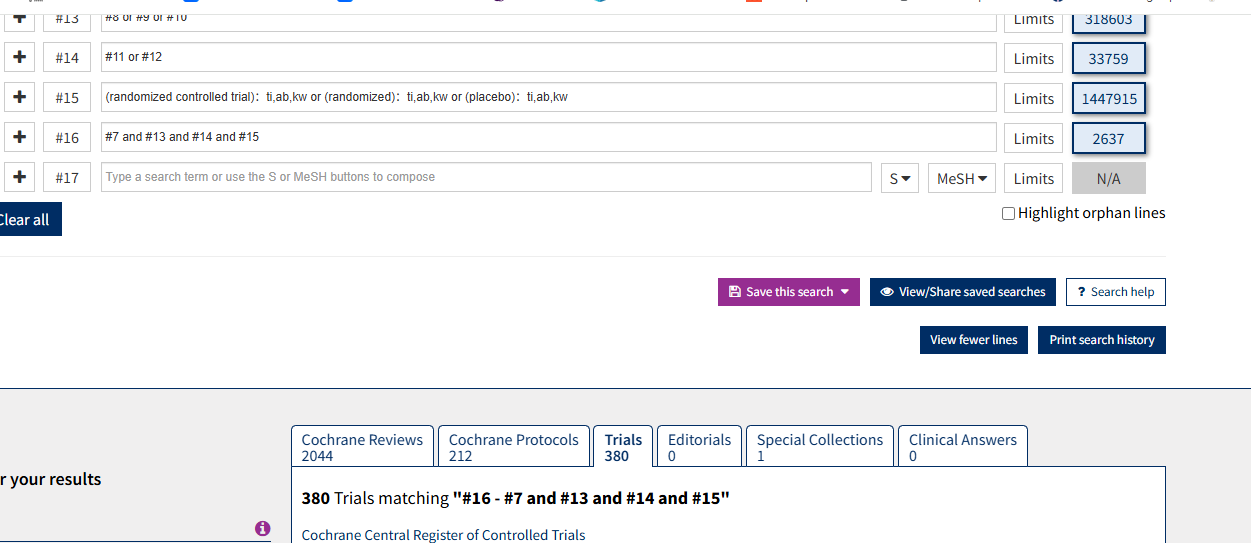

Supplement: Supplementary file 1 [file DataSheet2.zip › Retrieve records/2e0b95b6-7a21-435b-b4d8-a59d48e0c0ae.png]

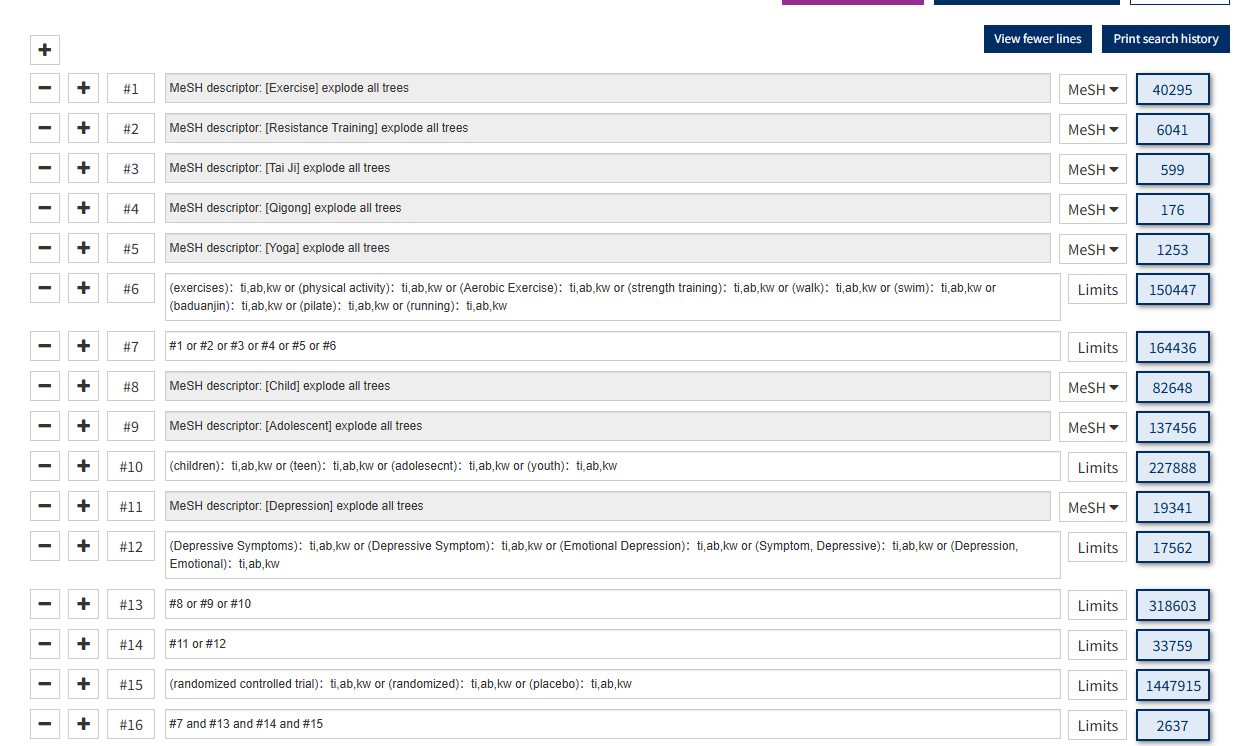

Supplement: Supplementary file 1 [file DataSheet2.zip › Retrieve records/31605a05-7b97-4627-9e1b-30cd991960d7.png]

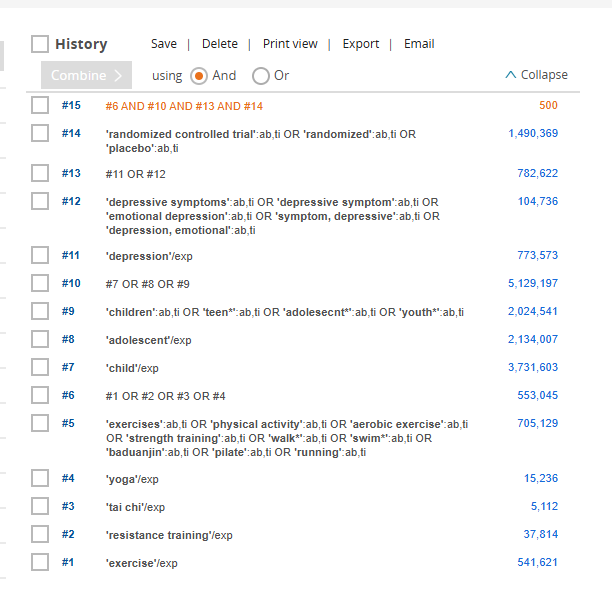

Supplement: Supplementary file 1 [file DataSheet2.zip › Retrieve records/58dc1c99-871d-442f-bd16-8da8df7ff6c3.png]

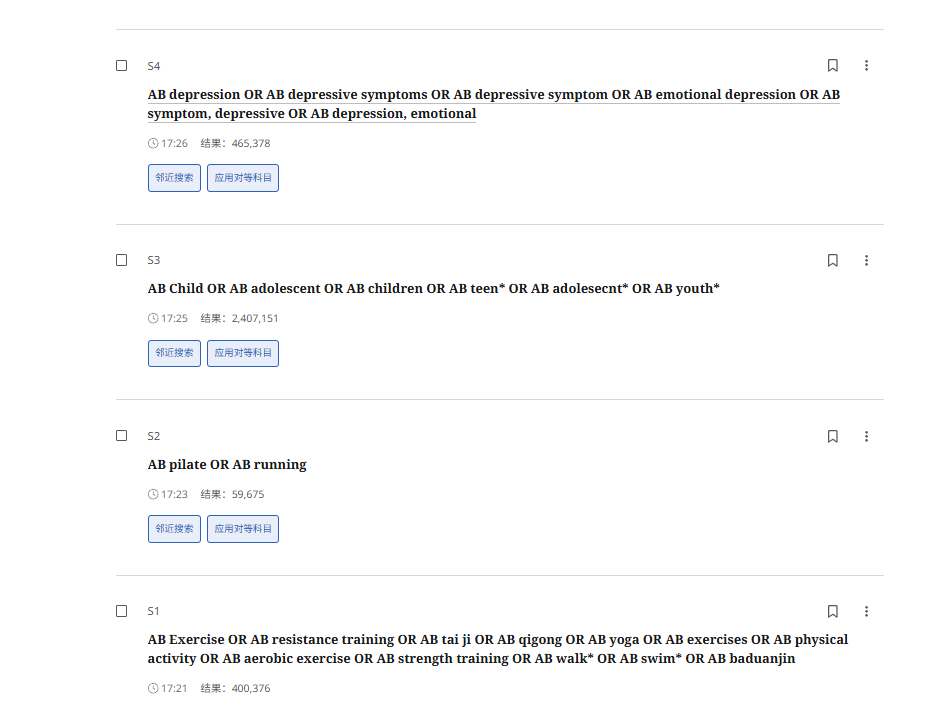

Supplement: Supplementary file 1 [file DataSheet2.zip › Retrieve records/a935f776-e07f-4939-bbbd-f97f8d12be1b.png]

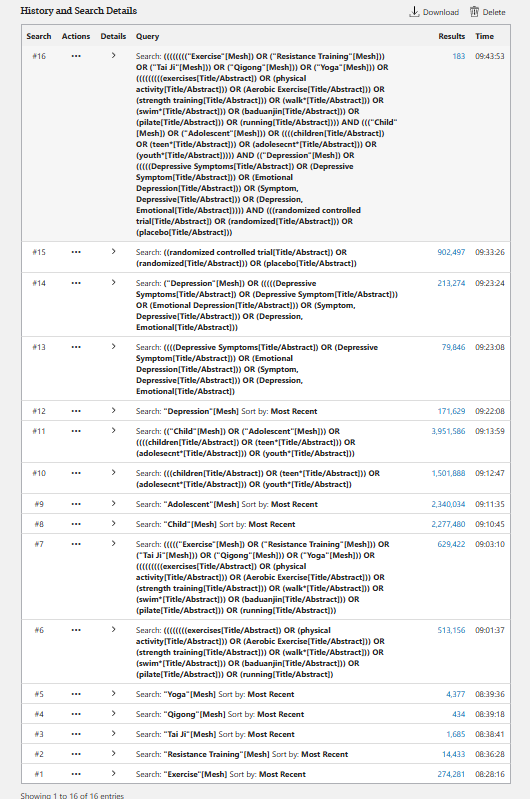

Supplement: Supplementary file 1 [file DataSheet2.zip › Retrieve records/b6346f3a-5c24-470a-b303-74af83fae326.png]

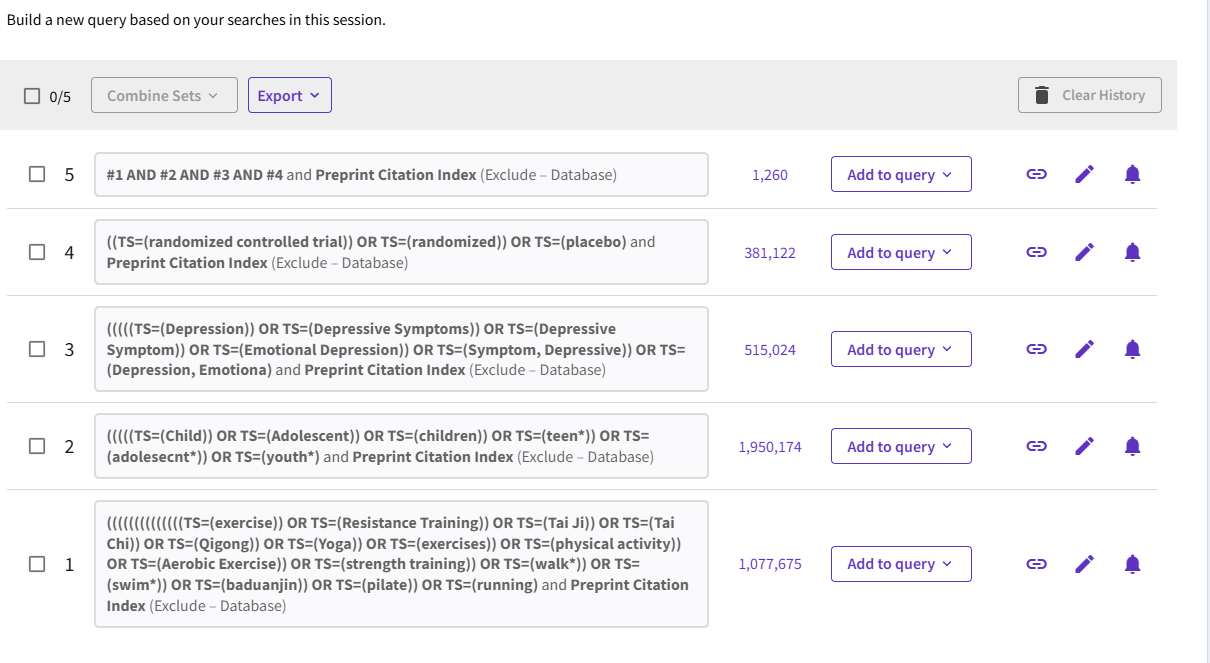

Supplement: Supplementary file 1 [file DataSheet2.zip › Retrieve records/ef78f0d3-d489-41a8-905e-82dad1412739.png]
